# Renal metabolism and hypertension

**DOI:** 10.1038/s41467-021-21301-5

**Published:** 2021-02-11

**Authors:** Zhongmin Tian, Mingyu Liang

**Affiliations:** 1 The Key Laboratory of Biomedical Information Engineering of Ministry of Education, School of Life Science and Technology, Xi’an Jiaotong University, Xi’an, Shaanxi China; 2 Center of Systems Molecular Medicine, Department of Physiology, Medical College of Wisconsin, Milwaukee, WI USA

**Keywords:** Hypertension, Kidney, Metabolism, Hypertension, Kidney

## Abstract

Hypertension is a leading risk factor for disease burden worldwide. The kidneys, which have a high specific metabolic rate, play an essential role in the long-term regulation of arterial blood pressure. In this review, we discuss the emerging role of renal metabolism in the development of hypertension. Renal energy and substrate metabolism is characterized by several important and, in some cases, unique features. Recent advances suggest that alterations of renal metabolism may result from genetic abnormalities or serve initially as a physiological response to environmental stressors to support tubular transport, which may ultimately affect regulatory pathways and lead to unfavorable cellular and pathophysiological consequences that contribute to the development of hypertension.

## Abstract (web summary)

Hypertension is one of the most important risk factors for cardiovascular disease. The kidneys, which have a very high metabolic rate, play a fundamental role in blood pressure regulation. In this review, the authors discuss recent studies on the role of renal metabolism in the development of hypertension.

## Introduction

Hypertension continues to be a leading risk factor for disease burden worldwide, despite the availability of several preventive and therapeutic approaches^1^. Hypertension substantially increases the risk of stroke, heart disease, chronic kidney disease, and cognitive decline^2,3^. Most hypertensive patients need to take antihypertensive medications continuously, as a cure is not available. Millions of patients remain hypertensive despite taking three or more antihypertensive medications^4^. Numerous genetic, epigenetic, lifestyle, and environmental factors may contribute to the development of hypertension. Understanding the physiological and molecular mechanisms underlying blood pressure regulation and utilizing this mechanistic understanding to sub-group hypertensive patients for precision prevention and treatment are important challenges in medical and biomedical research^3^.

Cardiac output and total peripheral vascular resistance determine systemic blood pressure. Several organs and tissues, including kidneys, resistance arterioles, central nervous system, and immune system, contribute to the regulation of blood pressure by regulating cardiac output or vascular resistance. The kidneys may regulate bodily fluid volume and vascular resistance by directly altering renal tubular transport of fluid and sodium or indirectly, by altering renal hemodynamics or endocrine factors^5,6^. Nearly all of the identified causal genes for Mendelian forms of human blood pressure abnormalities involve kidney function^7,8^, and most of the commonly used animal models of hypertension present kidney abnormalities^9^.

In addition to its essential fueling and housekeeping functions, intermediary metabolism is increasingly recognized for its regulatory role in which metabolic pathways and intermediate products influence gene expression, signal transduction, and other regulatory pathways in the cell^10^. Alterations in intermediary metabolism have been associated with the development of various conditions, including cancer and heart disease^11,12^. In the kidneys, intermediary metabolism and related cellular functions such as mitochondrial function play an essential role in the development of acute kidney injury and chronic kidney disease^13,14^.

Most of the energy produced in the kidneys is used to support renal tubular transport^15^, which is essential for the long-term regulation of blood pressure. Changes in renal energy and substrate metabolism may influence tubular transport by altering adenosine triphosphate (ATP) availability and levels of other metabolic intermediates with regulatory function. Therefore, renal energy and substrate metabolism might be important for regulating blood pressure and the development of hypertension. Moreover, energy and substrate metabolism might provide new interventional targets for the prevention or treatment of hypertension.

In this review, we provide a brief overview of renal metabolism and its association with tubular transport, summarize studies in humans and animal models that have examined renal energy and substrate metabolism in blood pressure regulation and hypertension, and outline challenges and opportunities in this exciting research area.

## Renal metabolism

The structure and function of the kidneys are highly compartmentalized. The primary functional unit of the kidneys is the nephron. The number of nephrons averages ~1 million in a human kidney. Each nephron consists of a glomerulus and a Bowman’s capsule connected serially to a proximal tubule, a loop of Henle, and a distal convoluted tubule, and several nephrons drain into a shared collecting duct.

**Fig. 1:**
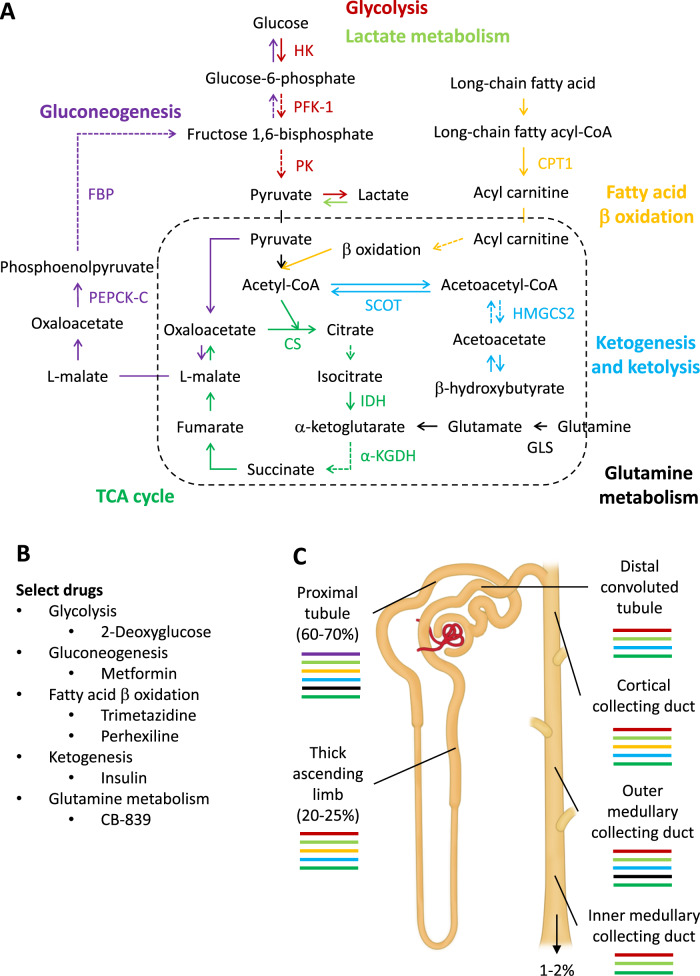
Renal metabolism and nephron segments. **A** Major metabolic pathways in the kidney. To make the figure comprehensible, only rate-limiting or key enzymes are shown, and arrows that contain multiple enzymatic steps are shown as dashed arrows. The box surrounded by the dashed line indicates mitochondria. FBP fructose 1,6-bisphosphatase, PEPCK-C cytosolic phosphoenolpyruvate carboxykinase, HK hexokinase, PFK-1 phosphofructokinase-1, PK pyruvate kinase, CPT1 carnitine palmitoyltransferase I, HMGCS2 mitochondrial HMG-CoA synthetase, SCOT succinyl-CoA:3-ketoacid-CoA transferase, GLS glutaminase, CS citrate synthase, IDH isocitrate dehydrogenase, α-KGDH α-Ketoglutarate dehydrogenase. **B** Examples of approved or investigational drugs targeting the metabolic pathways. **C** Major metabolic pathways in nephron segments. Colors of bars correspond to the colors of pathway names in panel A. The percentages indicate the percent of the filtered sodium reabsorbed or excreted.

A variety of substrates may be used as fuel in the kidneys. Major biochemical pathways relevant to renal substrate metabolism are summarized in Fig. 1A. Several of the metabolic pathways shown in Fig. 1A are targets of approved or investigational drugs. Prominent examples of these drugs and the pathways that they target are shown in Fig. 1B.

Renal metabolism is characterized by several important and, in some cases, unique features. Previous reviews have thoroughly described the intricate relation between renal metabolism and tubular transport^16–19^. The following section highlights salient points and recent studies in this area that are particularly relevant to understanding the role of renal metabolism in hypertension:

First, the kidneys have a high metabolic rate. The metabolic rate in the human kidneys has been estimated to be >400 kcal/kg tissue/day, which is the same as the heart, twice as high as the liver and the brain, and much higher than other organs^20^. Second, >80% of the oxygen consumed by the kidneys is used to support active transport machinery, primarily Na^+^/K^+^-ATPase located on the basolateral membrane of tubular cells^15^. Na^+^/K^+^-ATPase generates electrochemical gradients that directly or indirectly drive most of the remaining transport activities in the tubule. Third, blood flow and tissue oxygenation vary substantially between kidney regions. The renal cortex receives a blood flow that exceeds its metabolic needs but is necessary for the bulk filtration at glomeruli that is essential for removing whole-body metabolic wastes^21^. The partial pressure of oxygen (PO_2_) is ~50 mmHg in the renal cortex. Tissue PO_2_ decreases gradually into the renal medulla, reaching 10–15 mmHg in the renal inner medulla^19^. Fourth, substances used as fuel for energy may differ between the kidneys and other organs. For example, arterial−venous blood sampling and isotope tracing experiments in pigs indicate that circulating citrate contributes to the tricarboxylic acid (TCA) cycle most prominently in the kidneys and to an extent that is similar to glutamine and lactate^22^.

## Nephron segment metabolism and physiology

Each nephron segment has distinct physiological characteristics, and substrate utilization and metabolic pathway activities vary substantially between nephron segments and are generally consistent with oxygen availability (Fig. 1C). In regions where PO_2_ is high, nephrons use primarily oxidative phosphorylation to produce ATP, whereas segments where PO_2_ is low rely mainly on glycolysis. However, the current understanding of nephron segmental metabolism is mainly based on studies that measured specific substrate utilization, ATP production, and abundance or activities of a small number of metabolic enzymes in nephron segments isolated from rats, mice, and other animal models^17–19,23^. One should be cautious with extrapolating these findings to nephron segmental metabolism in vivo because metabolism is highly dynamic and dependent on the cellular milieu and anatomical context.

The proximal tubule reabsorbs ~65% of the filtered NaCl and water and nearly all filtered glucose and amino acids^21^. Part of this reabsorption may occur passively through the paracellular space^19^. Na^+^/K^+^-ATPase activity per unit length of the tubule segment and mitochondrial density and enzyme abundance in the proximal tubule are lower than or similar to the thick ascending limb of the loop of Henle and the distal convoluted tubule, but higher than other nephron segments^23^. Free fatty acids appear to be a significant energy source for the proximal tubule (Fig. 1C). Other substances that the proximal tubule may use as fuel include glutamine, lactate, and ketone bodies^17–19,23^. The proximal tubule has significant gluconeogenetic capabilities^17–19,23^. Gluconeogenesis may compete with Na^+^/K^+^-ATPase for ATP in the proximal tubule.

The thick ascending limb of the loop of Henle reabsorbs 20–25% of the filtered NaCl without reabsorbing water^21^. Glucose may be the primary energy source in thick ascending limb, even though lactate, fatty acids, and ketone bodies may also contribute. Glycolytic capabilities are present in the thick ascending limb and subsequent nephron segments and largely absent in the proximal tubule^17–19,23^. The thin descending and ascending limbs of the loop of Henle do not have significant active transport^21^.

The distal convoluted tubule and the collecting duct reabsorb 5–10% of the filtered sodium and are the final segments that may control sodium excretion and urine flow rate^21^. Substrate utilization in the cortical collecting duct is qualitatively similar to the thick ascending limb^17–19,23^. The importance of glucose as the main energy source appears to increase, and that of fatty acids decreases, as the collecting duct progresses to the renal inner medulla region.

Comprehensive transcriptome and proteome analyses have provided global views of mRNA and protein abundance of metabolic enzymes in kidney regions and nephron segments^24–27^, which are generally consistent with results of previous targeted analyses of enzyme activity, protein abundance, or substrate utilization.

## Role of renal metabolism in hypertension

### Renal metabolism in human hypertension

Tissue oxygenation levels are determined by oxygen supply and consumption and may reflect tissue metabolic activities. Oxygen consumption is determined by aerobic metabolism, which, in the kidneys, is largely determined by tubular transport activity. Oxygen supply to kidney tissue regions is determined by blood flow.

Renal regional tissue oxygenation levels in humans may be measured by blood oxygenation level-dependent magnetic resonance imaging (BOLD-MRI)^28^. A BOLD-MRI analysis in 10 normotensive subjects and eight untreated hypertensive patients indicated that a low-salt diet increased renal medullary tissue oxygenation levels in both groups when compared with a high-salt diet^29^. In the normotensive group, renal medullary oxygenation was correlated positively with proximal tubular reabsorption of sodium and negatively with distal sodium reabsorption. In another study examining patients with hypertension, renal medullary tissue oxygenation levels were significantly lower in a group of 20 African Americans compared with 29 Americans of European descent^30^. Furosemide, which inhibits sodium reabsorption in the thick ascending limb, increased medullary tissue oxygenation to similar levels in the two groups, suggesting that the thick ascending limb in African Americans might have greater reabsorptive activities and consume more oxygen^30^. The salt sensitivity level was not known for the individuals examined in this previous study; however, blood pressure in African Americans is more likely to be salt-sensitive compared with Americans of European descent^31^.

The Dietary Approaches to Stop Hypertension (DASH)-Sodium trial examined the effect of ~30 days of 50, 100, or 150 mmol of sodium intake on blood pressure using a randomized, crossover study design^32^. The trial demonstrated that higher sodium intake significantly increased blood pressure. A targeted metabolomic analysis identified a significant inverse correlation between urinary levels of β-aminoisobutyric acid (BAIBA), a metabolite of thymine and valine, and systolic blood pressure in a subset of DASH-Sodium subjects on the low- or high-sodium intake^33^. BAIBA was previously reported to be inversely correlated with cardiometabolic risk factors in the Framingham Heart Study cohort^34^. Positive correlations were identified for cystine, citrulline, homocysteine, and lysine with systolic blood pressure and cystine with diastolic blood pressure in the DASH-Sodium participants^33^. Urinary levels of several metabolites including fumarate, a TCA cycle intermediate, appeared to be able to classify the participants as salt-sensitive or salt-insensitive^33^.

In the absence of a change in glomerular filtration or tubular reabsorption and secretion of a metabolite, a dissociation of changes in urinary and plasma levels of the metabolite would suggest the intrarenal synthesis or catabolism of the metabolite has been altered. Renal handling of a metabolite, including intrarenal metabolism, may also influence plasma levels of the metabolite. Several studies have identified serum or plasma metabolites that are associated with blood pressure or hypertension or predictive of incident hypertension^35–37^. These metabolites include amino acids, such as glycine and serine, lactate, phospholipids, and fatty acids. The role of the kidneys in determining circulating levels of these metabolites and the effect of these metabolites on renal function remain to be examined.

### Genetic factors associated with intermediary metabolism and hypertension

Several DNA sequence variations that influence intermediary metabolism or mitochondrial function have been shown to contribute to the development of hypertension or are associated with blood pressure in humans. A homoplasmic mutation substituting cytidine for uridine immediately 5′ to the mitochondrial tRNA^Ile^ anticodon causes a cluster of maternally inherited diseases, including hypertension^38^. Mitochondrial tRNAs are required for the translation of proteins, including several components of the electron transport chain, encoded by the mitochondrial genome. Other mutations in mitochondrial tRNAs also reportedly cause maternally inherited hypertension, and these mutations decrease the efficiency of mitochondrial oxygen utilization^39^.

Genome-wide association studies involving as many as 1 million humans have identified >1000 genomic loci that are significantly associated with blood pressure^40,41^. The >26,000 single-nucleotide polymorphisms (SNPs) in these loci include nonsynonymous and potentially damaging SNPs in 63 genes^42^. In total, 12 of the 63 genes are known to be involved in intermediary metabolism or mitochondrial function (Table 1).

**Table 1:** Metabolism-related genes containing common amino acid sequence variations that are associated with human blood pressure^a^.

Gene symbol	Gene name
*ADO*	2-aminoethanethiol dioxygenase
*APOE*	Apolipoprotein E
*DBH*	Dopamine beta-hydroxylase
*DDHD2*	DDHD domain containing 2
*ERAP1*	Endoplasmic reticulum aminopeptidase 1
*F2*	Coagulation factor II, thrombin
*IMMT*	Inner membrane mitochondrial protein
*MTHFR*	Methylenetetrahydrofolate reductase
*PPRC1*	Peroxisome proliferator-activated receptor gamma, coactivator-related 1
*PRKAG1*	Protein Kinase AMP-activated non-catalytic subunit gamma 1
*PTPMT1*	Protein tyrosine phosphatase, mitochondrial 1
*RHOT2*	Ras homolog family member T2
*SULT1C3*	Sulfotransferase family 1C member 3
*TNXB*	Tenascin XB

^a^These genes are known to be involved in intermediary metabolism or mitochondrial function based on the gene functional annotation retrieved using the Database for Annotation, Visualization and Integrated Discovery (DAVID). In addition, these genes contain nonsynonymous and potentially damaging single-nucleotide polymorphisms associated with human blood pressure with genome-wide significance^42^.

Most of the blood pressure-associated SNPs are in noncoding regions of the genome and may influence blood pressure by influencing gene expression. An expression quantitative trait locus (eQTL) is a DNA sequence variant for which individuals possessing different alleles of the variant show different expression levels of a gene in one or more tissues^42^. Several hundred blood pressure-associated SNPs are eQTLs in kidney regional tissues or tissues included in the Genotype-Tissue Expression Project for 50 genes that are known to influence the physiology of blood pressure regulation^42^. In total, 23 of these 50 genes are known to be involved in intermediary metabolism or mitochondrial function (Table 2).

**Table 2:** Metabolism-related genes that may mediate the effect of common noncoding DNA sequence variations on human blood pressure^a^.

Gene symbol	Gene name	GTEx eQTL	eQTL kidney region
*ACE*	Angiotensin I converting enzyme	Yes	Glom, TI
*ADM*	Adrenomedullin	Yes	
*AGT*	Angiotensinogen	Yes	Glom, TI
*AVP*	Arginine vasopressin	Yes	
*CYP11B1*	Cytochrome P450 family 11 subfamily B member 1		Glom
*CYP4F12*	Cytochrome P450 family 4 subfamily F member 12		TI
*DDAH1*	Dimethylarginine dimethylaminohydrolase 1		TI
*DRD5*	Dopamine receptor D5		TI
*ENPEP*	Glutamyl aminopeptidase	Yes	
*ERAP1*	Endoplasmic reticulum aminopeptidase 1	Yes	Glom, TI
*ERAP2*	Endoplasmic reticulum aminopeptidase 2	Yes	Glom, TI
*GCH1*	GTP cyclohydrolase 1	Yes	
*LNPEP*	Leucyl and cysteinyl aminopeptidase	Yes	Glom
*LRP5*	LDL receptor-related protein 5	Yes	
*MME*	Membrane metalloendopeptidase		Glom
*NISCH*	Nischarin		Glom
*NOS3*	Nitric oxide synthase 3	Yes	
*NPPA*	Natriuretic peptide A	Yes	Glom
*NPR2*	Natriuretic peptide receptor 2	Yes	
*PDE4D*	Phosphodiesterase 4D		Glom
*PIK3R1*	Phosphoinositide-3-kinase regulatory subunit 1		TI
*SLC2A5*	Solute carrier family 2 member 5		TI
*TACR3*	Tachykinin Receptor 3		TI

*GTEx* Genotype-Tissue Expression Project, *eQTL* expression quantitative trait locus, *Glom* glomerulus, *TI* renal tubulointerstitial compartment.

^a^These genes are known to be involved in intermediary metabolism or mitochondrial function based on the gene functional annotation retrieved using the Database for Annotation, Visualization and Integrated Discovery (DAVID). In addition, these genes are eQTL genes for human blood pressure-associated single-nucleotide polymorphisms with genome-wide significance, and they are known to be physiologically important for blood pressure regulation based on Gene Ontology^42^.

The specific role of the kidneys in mediating the effect of these mitochondrial or nuclear DNA sequence variations and associated metabolic enzymes on blood pressure remains to be investigated. Hypertension is not an indication for renal biopsy, and hypertension often occurs together with other disease conditions, making it difficult to study the role of renal molecular or metabolic changes in the development of human hypertension. Nonetheless, a gene expression microarray analysis shows substantial downregulation of amino acid catabolism and synthesis, and fatty acid oxidation in kidneys biopsied from patients with hypertensive nephrosclerosis compared with healthy controls, which is associated with lower urine excretion of several amino acids^43^.

These aforementioned analyses performed in human subjects indicate that hypertension or blood pressure salt sensitivity is associated with changes in renal regional tissue oxygenation and energy and substrate metabolism, especially amino acid metabolism. Energy and substrate metabolism may contribute to the effect of rare and common genetic variants on blood pressure in humans.

### Renal metabolism in animal models of hypertension

Animal models are essential for hypertension research, since it is not possible to model blood pressure regulation adequately with any in vitro experimental system^44^. Renal metabolism has been studied in several animal models of hypertension, especially the Dahl salt-sensitive (SS) rat and the spontaneously hypertensive rat (SHR). The SS rat is the most widely used genetic model of human salt-sensitive hypertension^31^. SS rats exhibit a rapid and progressive increase of blood pressure within days upon exposure to a high-salt diet. The kidneys, including the renal medulla, play an essential role in the development of salt-induced hypertension in SS rats^45,46^. SHRs exhibit gradual and spontaneous increases in blood pressure with age.

Metabolic pathways are prominent findings of global, agnostic analyses of the kidneys from animal models of hypertension, similar to findings from human kidney biopsies with hypertensive nephrosclerosis^43^. An RNA-seq analysis of the renal outer medulla identified nine pathways that were altered between SS rats on a diet containing 0.4% salt and after seven days on the same diet containing 4% salt^24^. Seven of the nine pathways were involved in amino acid metabolism, and another was peroxisome proliferator-activated receptor (PPAR) signaling, which is a potent regulator of cellular metabolism. Another RNA-seq analysis of the renal outer medulla comparing SS rats on the 0.4% salt diet and after 14 days on the 4% salt diet identified eight pathways that included PPAR signaling and five pathways involved in amino acid metabolism^47^.

#### Oxygen metabolism and mitochondrial bioenergetics

Renal hypoxia may occur in hypertension and contribute to the development of hypertensive renal injury^48^. Whether changes in oxygen metabolism in the kidneys contribute to the development of hypertension is less clear. Renal oxygen metabolism is altered in SHR^49,50^. Renal inner medullary blood flow is reduced in prehypertensive SHR^51^. PO_2_ is significantly lower in the outer cortical proximal and distal convoluted tubules but not in the efferent arterioles of SHR when compared with normotensive Wistar Kyoto (WKY) rats^52^. Kidneys of SHR exhibit a sharp reduction in the oxygen utilization efficiency with significantly higher oxygen consumption for a unit of tubular sodium reabsorption^52^. Nitric oxide (NO) may decrease oxygen consumption by inhibiting several mitochondrial enzymes, including aconitase, electron transport chain complexes I and II, and cytochrome oxidase^53^. Stimulators of endogenous NO production decrease renal cortical oxygen consumption more prominently in WKY than they do in SHR^54^. This difference between SHR and WKY could be eliminated by the superoxide scavenger tempol. Basal oxygen consumption rate, ATP synthesis-linked oxygen consumption rate, and maximum and reserve respiration are higher in renal proximal tubule cells in primary culture from SHR^55^. Treatment with dichloroacetate, an inhibitor of pyruvate dehydrogenase kinase, increases pyruvate dehydrogenase activity and systolic blood pressure in 3–4 weeks old SHR and WKY rats^55^.

SS rats exhibit elevated reabsorption activities in the tubular loop that includes the thick ascending limb, which may contribute to the impaired pressure natriuresis in SS rats^56,57^. High-salt diet decreases cell surface Na^+^–K^+^−2Cl^−^ cotransporter (NKCC2) expression and furosemide-sensitive oxygen consumption, an index of NKCC2-sensitive sodium reabsorption, in the thick ascending limb of salt-resistant (SR) rats but not in SS rats^58^. Renal medullary blood flow is decreased in SS rats within a few days after the start of a high-salt diet^59,60^.

**Fig. 2:**
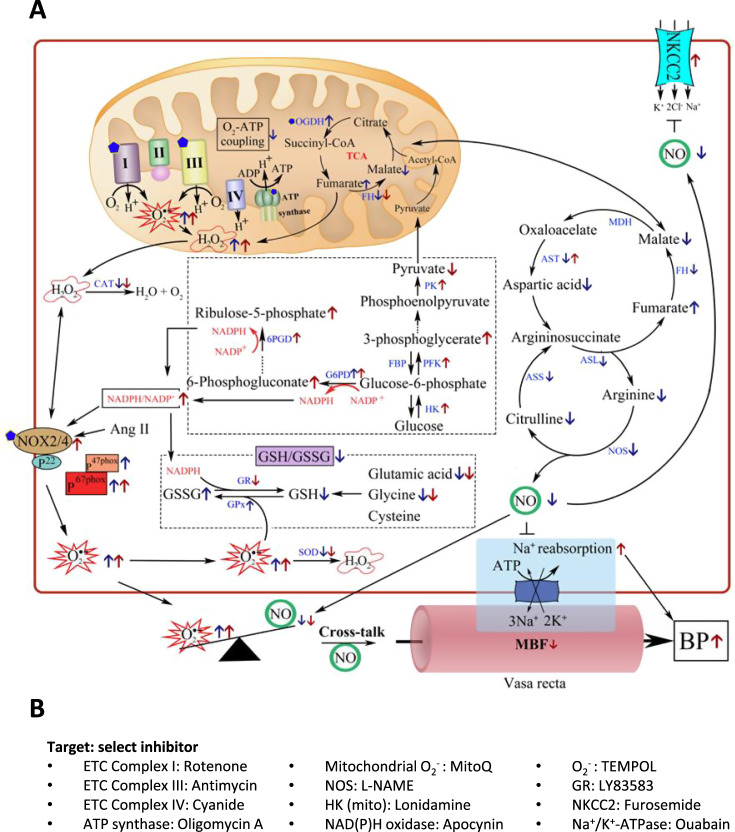
Renal metabolic mechanisms of hypertension in Dahl salt-sensitive (SS) rats. **A** Renal metabolic mechanisms of hypertension. This figure is primarily based on data obtained from analyses of the renal medulla or the medullary thick ascending limb of the loop of Henle in SS rats. Some of the indicated changes also occur in the renal cortex. The blue arrows represent increased or decreased content (or activity) in SS rats relative to salt-insensitive rats, including SS-13^BN^ rats. The red arrows represent increased or decreased content (or activity) in SS rats fed a high-salt diet. Blue pentagons represent sites of reactive oxygen species generation. Black arrow and inverted T mark (⊥) represent positive and negative influence, respectively. Some of these regulatory relations and their causal role in the development of hypertension remain to be tested in the specific context of SS kidneys. TCA tricarboxylic acid cycle, OGDH α-ketoglutarate dehydrogenase, FH, fumarase; MDH, malic dehydrogenase; ASS, argininosuccinate synthetase; ASL, argininosuccinate lyase; AST, aspartate aminotransferase, NOS nitric oxide synthase, HK hexokinase, PFK phosphofructokinase, G6PD glucose-6-phosphate dehydrogenase, 6GPD 6-phosphogluconate dehydrogenase, GSH glutathione, GSSG glutathione disulfide, GR glutathione reductase, GPx glutathione peroxidase, NKCC2 Na–K-2Cl cotransporter, SOD superoxide dismutase, CAT catalase, MBF medullary blood flow. **B** Examples of chemical inhibitors of components of the metabolic mechanisms shown in panel (**A**). Other inhibitors may be available, and the inhibitors listed may have additional targets. ETC electron transport chain, L-NAME L-N^G^-nitro arginine methyl ester.

Mitochondrial alterations have been reported in the kidneys of SS rats (Fig. 2). Longer mitochondria (>2 μm), which may indicate healthier mitochondria, account for a significantly smaller fraction of mitochondria in medullary thick ascending limbs of the loop of Henle, but a larger fraction in proximal tubules, in SS rats compared with salt-insensitive consomic SS.13^BN^ rats and Sprague-Dawley (SD) rats^61^. These changes occur before the development of substantial hypertension and overt renal injury. The oxygen consumption rate of intact medullary thick ascending limb cells and state 3 respiration of mitochondria isolated from the renal outer medulla are lower in SS rats than in SS.13^BN^ rats fed an 8% NaCl diet for 7 days^62^. Proteomic analysis of mitochondria isolated from medullary thick ascending limbs identified several proteins as differentially expressed between the two rat strains^62^. ATP contents of mitochondria isolated from the renal cortex or medulla are similar between SS and SS.13^BN^ rats on a 0.4% salt diet, while mitochondrial membrane potential and ATP production rate are lower in SS rats^63^. Treatment with a 4% NaCl diet for 7 or 21 days resulted in lower ratios of ATP/ADP, GTP/GDP, and NADH/NAD^+^ in the glomeruli, but not the cortical tissue, of SS rats^64^. These studies suggest structural and functional changes might occur in mitochondria in kidneys of hypertension models.

Changes in renal oxygen metabolism or mitochondrial bioenergetics may lead to changes in the level of substrate metabolic intermediaries, which in turn influence blood pressure regulation, as discussed in later sections of this article. Changes in renal oxygen metabolism and mitochondrial bioenergetics may also lead to changes in reactive oxygen species (ROS) production (Fig. 2). Excessive ROS, particularly superoxide and hydrogen peroxide, is present in the kidneys of animal models of hypertension and may contribute to the development of hypertension through several mechanisms, such as decreasing NO bioavailability^46,65^. NADP(H) oxidase is upregulated and ROS scavenging enzymes superoxide dismutase and catalase are downregulated in the kidneys of SS rats on a high-salt diet^66–68^. In mitochondria, electron “leaks” from the electron transport chain may lead to a one-electron reduction of O_2_ and the generation of superoxide^69–71^. Mitochondrial ROS may contribute to the development of hypertension and mitochondria-targeted antioxidants may attenuate hypertension^72–76^. Uncoupling proteins (UCP) allow proton leakage back across the inner mitochondrial membrane without generating ATP and could lower oxygen utilization for ATP production and increase oxygen consumption. Mice null for the redox-sensitive chaperone DJ-1 exhibit hypertension and an upregulation of renal UCP2 expression. UCP2 knockdown by renal subcapsular infusion of a siRNA attenuates hypertension and increases serum NO metabolite levels in these mice^77^. How the changes in renal oxygen metabolism and mitochondrial bioenergetics in hypertensive animal models may change mitochondrial ROS production remains to be investigated.

#### The TCA cycle

Proteomic analysis of the renal cortex and medulla identified fumarase as one of the proteins that showed the most substantial differences in expression between SS and SS.13^BN^ rats^26^. Fumarase converts fumarate to L-malate in the TCA cycle. The gene that encodes fumarase, *Fh*, contains a nucleotide difference between the SS allele and the BN allele that results in the presence of lysine at amino acid position 481 in SS rats and glutamic acid in BN and SS-13^BN^ rats^78^. Despite an apparent compensatory increase in fumarase abundance in SS rats, total fumarase activity in the kidneys is significantly lower in SS rats compared with SS.13^BN^ rats^78,79^. Transgenic overexpression of fumarase in SS rats attenuates salt-induced hypertension^80^. The knockdown of renal fumarase in SD rats using a siRNA delivered directly into the renal medullary interstitium exacerbates salt-induced hypertension^80^. Intravenous infusion of a fumarate precursor in SS.13^BN^ rats results in a fumarate excess in the renal medulla comparable with that seen in SS rats and significantly exacerbates salt-induced hypertension in SS.13^BN^ rats^78^. Renal medullary H_2_O_2_ contributes to the development of salt-induced hypertension in SS rats^81^. Fumarate increases the levels of H_2_O_2_ in the renal medulla in vivo and cultured human renal epithelial cells in vitro, the mechanism of which remains unclear^78,82^. NADPH oxidase-derived ROS may downregulate fumarase and increase fumarate in mouse glomeruli^83^, potentially forming a vicious cycle between fumarate and ROS.

L-malate is converted to oxaloacetate by malate dehydrogenase. Oxaloacetate may be combined with acetyl-CoA to form citrate in the TCA cycle but can also be converted to aspartate via aspartate transaminase. Aspartate may be combined with citrulline by argininosuccinate synthase to form argininosuccinate, which is converted to L-arginine and fumarate by argininosuccinate lyase. L-arginine is the substrate for NO synthase (NOS) for the generation of NO and citrulline. Renal NO protects against the development of hypertension through its vasodilatory effect as well as direct inhibition of sodium reabsorption at several nephron segments^84,85^. L-arginine, given systemically or directly into the renal interstitium, substantially attenuates hypertension in SS rats^86,87^. Aspartate, citrulline, L-arginine, and NO levels are reduced in the kidneys of SS rats compared with SS.13^BN^ rats^79^. Oral supplementation with L-malate or aspartate in SS rats reverses the reduction of these metabolites in the kidneys and attenuates salt-induced hypertension^79^. A heterozygous mutation in *Nos3* that results in haploinsufficiency of eNOS exacerbates hypertension and renal injury in SS rats. Transgenic overexpression of fumarase in these rats increases NO and the ratio of L-arginine/citrulline in the renal outer medulla and abolishes hypertension and renal injury that may be attributed to *Nos3* heterozygous mutation^88^. In addition, oral malate supplementation attenuates high-salt-induced elevation of H_2_O_2_ and lipid peroxidation in the renal medulla of SS rats^82^.

These findings suggest that fumarase insufficiency in SS rats might contribute to the predisposition to developing salt-sensitive hypertension by decreasing NO and increasing H_2_O_2_ in the kidneys (Fig. 2). In salt-insensitive humans or animals, a high-salt diet may elicit adaptive responses in renal metabolism that prevent the development of hypertension. Pre-existing defects in salt-sensitive individuals, such as fumarase insufficiency, may hinder such adaptive responses to high-salt intake, resulting in the development of hypertension.

Other TCA cycle components in the kidneys also may be involved in the regulation of blood pressure. Intravenous injection of succinate into rats and mice induces hypertension via activation of the renin–angiotensin system, and this response is abolished in GPR91-deficient mice^89^. The activation of succinate receptor GPR91 could trigger renin release from macula densa cells in the distal convoluted tubule^90,91^. Circulating succinate is associated with the elevation of blood pressure in SHR^92^. Succinate increases in the plasma, but not in the renal medulla of SS rats, compared with SS.13^BN^ rats^78,93^.

DNA methylation and demethylation in the renal medulla are involved in the development of hypertension in SS rats^94,95^. DNA demethylation catalyzed by ten-eleven translocase requires α-ketoglutarate. Circulating citrate may be a significant source of energy in the kidney^22^. Despite these advances, the precise role of these TCA cycle intermediates in the kidneys in the development of hypertension remains to be investigated.

#### Carbohydrate metabolism

The proximal tubule normally has low, if any, glycolytic activity^23,25^. However, proximal tubule cells in primary culture from SHR showed a higher extracellular acidification rate than cells from WKY rats, suggesting elevated glycolytic activity and capacity in SHR^55^. Lactate levels in renal cortical homogenate are slightly higher in SHR than WKY^55^.

Several metabolites and enzymes in the glycolysis and pentose phosphate pathways of glucose catabolism, including 3-phosphoglycerate, 6-phosphogluconate, and ribulose-5-phosphate, are elevated in the kidneys of SS rats fed a high-salt diet (Fig. 2)^82,96^. The pentose phosphate pathway produces NADPH from NADP. The NADPH/NADP ratio is higher in SS rats fed a high-salt diet^96^. NADPH is a limiting factor for the activity of NADPH oxidase that produces superoxide, and 6-phosphogluconate dehydrogenase may directly interact with the NADPH oxidase complex^97–99^.

Methylglyoxal (MG) may be produced as a side product of glycolysis. MG could react with lysine, arginine, and cysteine residues of proteins to form irreversible advanced glycation end products^100^. Plasma and renal levels of MG and renal levels of MG-induced advanced glycation end products were higher in SHR than WKY rats^101^. MG increases blood pressure and exacerbates renal injury and oxidative stress in SS rats on a 1% NaCl diet, and these effects were attenuated by the angiotensin II receptor blocker candesartan^102^.

High plasma levels of insulin may contribute to hypertension by stimulating renal tubular sodium reabsorption^103,104^. SS rats exhibit signs of insulin resistance^105^. Whether this insulin resistance contributes to sodium retention or hypertension in SS rats is not clear. Fasting plasma glucose and plasma insulin levels, renal insulin receptor mRNA levels, and insulin-binding parameters are similar between SS and SR rats fed either low- or high-salt chow^105,106^. Notably, the mechanisms underlying insulin resistance in SS rats did not appear to involve canonical insulin signaling^107^.

#### Amino acid metabolism

Systemic changes in amino acid levels are associated with hypertension, and fluid and sodium homeostasis. Lower plasma levels of a large number of amino acids were observed in a group of young hypertensive men compared with control^36^. A combined treatment of a high-salt diet with saline drinking in mice causes broad changes in energy and substrate metabolism in the liver and skeletal muscle, including amino acid catabolism in the muscle^108^. SS rats exhibit significant changes in plasma amino acid levels and skeletal muscle amino acid metabolism compared with SS.13^BN^ rats or in response to a high-salt diet, especially glycine, serine, and threonine metabolism and alanine, aspartate, and glutamate metabolism^93,109^. Serum levels of metabolites, including several amino acids and TCA cycle intermediates, have been reported to show circadian variation patterns that may be different between SHR and WKY rats^110^.

Renal metabolism of several amino acids may contribute to the development of hypertension by influencing blood pressure regulatory mechanisms. The relation of these amino acids with renal energy metabolism is largely unclear as amino acids, with the exception of glutamine, are not normally a key source of energy in the kidneys. However, it is possible that amino acids are used as fuel in the kidneys when renal metabolic abnormalities occur.

The antihypertensive effect of L-arginine, likely through enhancing NO production, is well-established in animal models. NO production and endothelial NOS expression are decreased in SHR compared with WKY^111–113^. Perinatal dietary supplementation with L-arginine and antioxidants reduces blood pressure in SHR^114^. L-arginine alone, however, may not attenuate hypertension in SHR^87,115^. Renal levels of L-arginine and NO are lower in SS rats^79^. NOS activities in the renal outer medulla are lower in SS rats compared with SS.13^BN^ rats after six weeks of a high-salt diet^66^. Activities of neuronal NOS are lower in SS rats than SR rats after four weeks of a high-salt diet^116^. L-arginine, administered through renal medullary interstitial infusion^86^, intravenous infusion^117,118^, intraperitoneal injections^87^ or oral supplementation^87,119,120^, increases the generation of NO and substantially attenuates hypertension in SS rats.

Renal L-arginine may come from the endogenous synthesis in the kidneys or circulating L-arginine. Circulating L-arginine mainly comes from intestinal absorption of protein-derived L-arginine and free L-arginine in the food^121^. Endogenous L-arginine is mainly synthesized in the liver and kidneys through the urea cycle. L-arginine synthesized in the liver does not reach the systemic circulation effectively because of the high activity of hepatic arginase^122,123^. The lower level of renal L-arginine in SS rats might result, in part, from fumarase insufficiency and the subsequent reduction of L-arginine regeneration from citrulline and aspartate, as discussed earlier in this article (Fig. 2). L-arginine transport, which may be inhibited competitively by L-lysine, also appears to be involved in angiotensin II-induced renal cortical vasoconstriction in SD rats and low renal NO bioavailability in SHR^113,124^.

Citrulline and aspartate are the substrates of endogenous L-arginine synthesis in the kidney. Citrulline is a nonessential amino acid mainly derived from intestinal glutamine breakdown. The liver does not take up citrulline^125–127^; however, the kidneys may take up circulating citrulline and convert it to L-arginine. Argininosuccinate synthase is a rate-limiting enzyme in the citrulline-NO cycle, and its expression and activity can be induced by citrulline^128^. Citrulline improves renal NO levels and attenuates hypertension in SS and SHR rats^87,129,130^.

The metabolism of glycine, glutamate, and cysteine may be involved in the development of hypertension by influencing the homeostasis of glutathione (GSH), an important antioxidant, and glutathione disulfide (GSSG) (Fig. 2). The synthesis of GSH is regulated by cysteine availability and GSH/GSSG feedback inhibition^131^. Cysteine, delivered as its stable analog N-acetyl cysteine, has antihypertensive effects in humans and animal models and may work directly or through its storage form GSH to decrease oxidative stress^132^. Levels of glycine and glutamate in the renal medulla are lower in SS rats compared with SS.13^[BN 82^. The ratio of GSH/GSSG is lower in the kidney, particularly the renal medulla, of SS rats compared with SS.13^[BN 82^. Glutathione reductase is downregulated and glutathione peroxidase upregulated in the kidneys of SS rats on a high-salt diet^66,96^.

The kidneys influence the body pool of another cysteine-related amino acid, taurine, by regulating tubular reabsorption of taurine^133^. Taurine attenuates hypertension in humans and several animal models, including SS rats and SHR^134–137^. Taurine reduces oxidative stress and elevates kallikrein in the kidney.

Catecholamines, including dopamine, norepinephrine, and epinephrine, play a significant role in regulating renal hemodynamics, renal tubular transport, and blood pressure. Catecholamines are metabolic products of the amino acid tyrosine. Renal proximal tubules and possibly the distal nephron may take up the tyrosine product 3,4-dihydroxyphenylalanine and convert it to dopamine^138^.

Urinary levels of BAIBA, a nonprotein amino acid produced by catabolic metabolism of thymine or branched-chain amino acid valine, are inversely correlated with systolic blood pressure in humans on low- and high-sodium intakes as discussed earlier in this article^33^. Treatment with BAIBA significantly attenuates salt-induced hypertension in SS rats^33^. Alanine-glyoxylate aminotransferase-2 (AGXT2) is one of the enzymes involved in the metabolism of BAIBA. AGXT2 also may degrade asymmetric dimethylarginine, an endogenous inhibitor of NOS. AGXT2 knockout mice exhibit increased asymmetric dimethylarginine and reduced NO and develop hypertension^139^. Treatment of SS rats with a high-salt diet downregulates valine and another branched-chain amino acid leucine in glomeruli^64^.

The amount and source of dietary protein influence the development of hypertension^47,140,141^. It remains to be investigated whether changes in renal metabolism, including amino acid metabolism, contribute to the effect of dietary protein on the development of hypertension.

#### Lipid metabolism

Obesity may contribute to the development of hypertension by altering the renal function through the activation of the sympathetic nervous system and the renin–angiotensin–aldosterone system^142^. Obesity is associated with abnormalities in bioenergetics in several organ systems, and β oxidation of fatty acids, a major fuel for the kidney, has been implicated in the development of renal injury. However, the role of renal bioenergetic metabolism of lipids in the development of hypertension is largely unclear.

Blood pressure, renal tissue content of triglycerides, and lipid droplets in tubular cells are greater in Otsuka Long-Evans Tokushima Fatty rats than Long-Evans Tokushima Otsuka rats. Treatment with a calcium channel blocker, benidipine, or an angiotensin type 1 receptor blocker, losartan, decreases blood pressure, reduces lipid accumulation in the kidneys, and increases the expression of carnitine palmitoyltransferase-1^143^. Alport syndrome mice develop hypertension and exhibit cholesterol accumulation, dynamin-3 and LDL receptor upregulation, and defective mitochondria in the renal tubule^144^. Osteopontin gene deletion reduces renal expression of dynamin-3 and LDL receptor and lowers blood pressure in Alport syndrome mice^144^.

A high-salt diet leads to a decrease in the serum level of the ketone body β-hydroxybutyrate in fasting SS rats. Nutritional supplementation of β-hydroxybutyrate precursor, 1,3-butanediol, attenuates renal inflammation and hypertension in SS rats^145^. It has been suggested that the cardiovascular and renal benefits of sodium-glucose cotransporter 2 (SGLT2) inhibitors might be in part because the inhibitors cause a shift in myocardial and renal fuel metabolism from fat and glucose oxidation to ketone bodies^146^. It is unclear whether any such shift is relevant to the blood pressure-lowering effect of SGLT2 inhibition.

Non-energetic metabolism of lipids in the kidneys produces several metabolites that play significant roles in the regulation of blood pressure through their effects on renal hemodynamics and tubular transport. These metabolites include cytochrome P450 metabolites of arachidonic acids 20-hydroxyeicosatetraenoic acid and epoxyeicosatrienoic acids, cyclooxygenase metabolites prostaglandin E2, prostaglandin I2, and thromboxane A2, and lipoxygenase metabolites leukotrienes, hydroxyeicosatetraenoic acids, and lipoxins. The role of these metabolites in the development of hypertension has been reviewed elsewhere^147–149^.

## Summary and perspectives

**Fig. 3:**
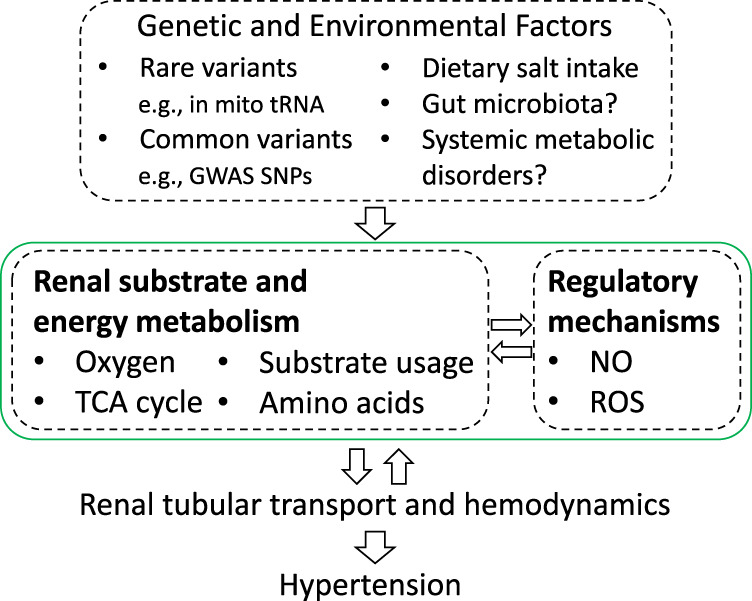
The proposed overall mechanism by which renal energy and substrate metabolism contribute to the development of hypertension. It is well-established that genetic and environmental factors influence renal tubular transport and hemodynamics, which, in turn, contribute to the development of hypertension and cause changes in renal energy and substrate metabolism. Recent advances in human and animal model research indicate that renal energy and substrate metabolism may also influence the development of hypertension, which may be mediated by novel effects of renal energy and substrate metabolism on regulatory substances including NO and ROS and subsequent effects on renal tubular transport and hemodynamics. Mito mitochondria, GWAS SNPs blood pressure-associated single-nucleotide polymorphisms identified by genome-wide association studies, TCA tricarboxylic acid, NO nitric oxide, ROS reactive oxygen species.

In summary, recent studies have led to several key advances in our understanding of the role of renal energy and substrate metabolism in the development of hypertension (Fig. 3). First, several rare and common genetic variants that influence blood pressure in humans may do so by affecting energy or substrate metabolism. Second, hypertension or blood pressure salt sensitivity is associated with changes in renal tissue oxygenation and substrate metabolism, especially amino acid metabolism, in both humans and well-established animal models. Third, renal energy and substrate metabolism may influence the development of hypertension via a range of mechanisms, some unexpected. For example, TCA cycle enzymes or intermediaries may influence hypertension by changing the level of amino acids, NO or ROS or binding to orphan receptors^78,79,88,89^.

Renal energy and substrate metabolism are closely tied to renal hemodynamics and tubular transport. Changes in renal tubular transport or hemodynamics may alter energy demands or oxygen supply, leading to changes in renal energy metabolism. Emerging evidence reviewed in this article suggests that the reverse might also occur (Fig. 3). That is, alterations of renal energy and substrate metabolism may influence renal tubular transport and hemodynamics and thereby the regulation of blood pressure and the development of hypertension. These alterations of renal energy and substrate metabolism may result from inherent abnormalities, including genetic defects, attempts of the kidneys to respond to environmental stressors, such as high-salt intake, or a combination of internal and external factors. The alterations of renal energy and substrate metabolism may meet the energy demand but disturb regulatory mechanisms, such as NO levels and redox balance, resulting in dysregulation of renal tubular transport and hemodynamics and the development of hypertension. It is an intriguing possibility that renal energy and substrate metabolism may influence blood pressure through mechanisms that are not dependent on bioenergetics alone.

Thorough investigation of the regulatory model shown in Fig. 3 requires concerted efforts of physiologists, biochemists, geneticists, and computational biologists and a molecular systems medicine approach^94,150,151^. Going forward, it will be of primary importance to better understand the in vivo metabolic profiles and dynamics in the kidneys and nephron segments of animals and humans, and the investigation of genetic and environmental factors that lead to the development of hypertension by influencing these metabolic processes may help identify any pro-hypertensive regulatory dysfunctions that result from such metabolic abnormalities. Ultimately, it will be important to examine whether targeting these metabolic abnormalities may represent an advantageous therapeutic approach for certain subgroups of hypertensive patients. Recent studies have begun to shed light on these questions, but the study of the role of renal energy and substrate metabolism in the development of hypertension remains a largely open field.

Several exciting areas of research provide additional opportunities to explore the role of renal energy and substrate metabolism in hypertension (Fig. 3). Obesity, diabetes, and other systemic metabolic disorders are closely related to hypertension. New treatments for diabetes, such as SGLT2 inhibitors, have significant blood pressure-lowering effects^152^. Changes in the gut microbiota have also been shown to influence blood pressure^153^. It would be interesting to understand how broad metabolic disturbances in patients with systemic metabolic disorders or altered gut microbiota may involve renal energy and substrate metabolism and whether the renal metabolic involvement might play a role in the development and progression of hypertension in these patients.

## References

[1] GBD 2017 Risk Factor Collaborators (2018). Global, regional, and national comparative risk assessment of 84 behavioural environmental and occupational, and metabolic risks or clusters of risks for 195 countries and territories, 1990–2017: a systematic analysis for the Global Burden of Disease Study 2017. Lancet.

[2] Launer LJ, Masaki K, Petrovitch H, Foley D, Havlik RJ (1995). The association between midlife blood pressure levels and late-life cognitive function. The Honolulu-Asia Aging Study. Jama.

[3] Kotchen TA, Cowley AW, Liang M (2016). Ushering hypertension into a new era of precision medicine. Jama.

[4] Carey RM (2018). Resistant hypertension: detection, evaluation, and management: a scientific statement from the American Heart Association. Hypertension.

[5] Cowley AW (1992). Long-term control of arterial blood pressure. Physiological Rev..

[6] Guyton AC (1991). Blood pressure control–special role of the kidneys and body fluids. Science.

[7] Lifton RP, Gharavi AG, Geller DS (2001). Molecular mechanisms of human hypertension. Cell.

[8] Ehret, G. B. *Genetic Factors in the Pathogenesis of Hypertension*, https://www.uptodate.com/contents/genetic-factors-in-the-pathogenesis-of-hypertension (2017).

[9] Cowley AW, Roman RJ (1996). The role of the kidney in hypertension. Jama.

[10] McKnight SL (2010). On getting there from here. Science.

[11] Vander Heiden MG, DeBerardinis RJ (2017). Understanding the intersections between metabolism and cancer biology. Cell.

[12] Bertero E, Maack C (2018). Metabolic remodelling in heart failure. Nat. Rev. Cardiol..

[13] Bhargava P, Schnellmann RG (2017). Mitochondrial energetics in the kidney. Nat. Rev. Nephrol..

[14] Forbes JM, Thorburn DR (2018). Mitochondrial dysfunction in diabetic kidney disease. Nat. Rev. Nephrol..

[15] Mandel LJ, Balaban RS (1981). Stoichiometry and coupling of active transport to oxidative metabolism in epithelial tissues. Am. J. Physiol..

[16] Lewy PR, Quintanilla A, Levin NW, Kessler RH (1973). Renal energy metabolism and sodium reabsorption. Annu. Rev. Med..

[17] Mandel LJ (1985). Metabolic substrates, cellular energy production, and the regulation of proximal tubular transport. Annu. Rev. Physiol..

[18] Soltoff SP (1986). ATP and the regulation of renal cell function. Annu. Rev. Physiol..

[19] Singh, P., Thomson, S. C. & McDonough, A. A. in *Brenner and Rector’s The Kidney* 11th edn (2019).

[20] Elia, M. in *Energy Metabolism: Tissue Determinants and Cellular Corollaries* (Raven Press, Ltd., 1992).

[21] Hall, J. E. *Guyton and Hall Textbook of Medical Physiology* 13th edn (2015).

[22] Jang C (2019). Metabolite exchange between mammalian organs quantified in pigs. Cell Metab..

[23] Guder WG, Ross BD (1984). Enzyme distribution along the nephron. Kidney Int..

[24] Liu Y, Liu P, Yang C, Cowley AW, Liang M (2014). Base-resolution maps of 5-methylcytosine and 5-hydroxymethylcytosine in Dahl S rats: effect of salt and genomic sequence. Hypertension.

[25] Lee JW, Chou CL, Knepper MA (2015). Deep sequencing in microdissected renal tubules identifies nephron segment-specific transcriptomes. J. Am. Soc. Nephrology: JASN.

[26] Tian Z (2008). Renal regional proteomes in young Dahl salt-sensitive rats. Hypertension.

[27] Rinschen MM, Limbutara K, Knepper MA, Payne DM, Pisitkun T (2018). From molecules to mechanisms: functional proteomics and its application to renal tubule physiology. Physiological Rev..

[28] Prasad PV, Edelman RR, Epstein FH (1996). Noninvasive evaluation of intrarenal oxygenation with BOLD MRI. Circulation.

[29] Pruijm M (2010). Effect of sodium loading/depletion on renal oxygenation in young normotensive and hypertensive men. Hypertension.

[30] Textor SC (2012). Association of filtered sodium load with medullary volumes and medullary hypoxia in hypertensive African Americans as compared with whites. Am. J. Kidney Dis..

[31] Kotchen TA, Cowley AW, Frohlich ED (2013). Salt in health and disease—a delicate balance. N. Engl. J. Med..

[32] Sacks FM (2001). Effects on blood pressure of reduced dietary sodium and the Dietary Approaches to Stop Hypertension (DASH) diet. DASH-Sodium Collaborative Research Group. N. Engl. J. Med..

[33] Cheng Y (2018). Urinary metabolites associated with blood pressure on a low- or high-sodium diet. Theranostics.

[34] Roberts LD (2014). beta-Aminoisobutyric acid induces browning of white fat and hepatic beta-oxidation and is inversely correlated with cardiometabolic risk factors. Cell Metab..

[35] Menni C (2015). Metabolomic identification of a novel pathway of blood pressure regulation involving hexadecanedioate. Hypertension.

[36] Wang L (2015). Reconstruction and analysis of correlation networks based on GC-MS metabolomics data for young hypertensive men. Analytica Chim. Acta.

[37] Dietrich S (2016). Identification of serum metabolites associated with incident hypertension in the European Prospective Investigation into Cancer and Nutrition-Potsdam Study. Hypertension.

[38] Wilson FH (2004). A cluster of metabolic defects caused by mutation in a mitochondrial tRNA. Science.

[39] Wang S (2011). Maternally inherited essential hypertension is associated with the novel 4263A>G mutation in the mitochondrial tRNAIle gene in a large Han Chinese family. Circulation Res..

[40] Giri A (2019). Trans-ethnic association study of blood pressure determinants in over 750,000 individuals. Nat. Genet..

[41] Evangelou E (2018). Genetic analysis of over 1 million people identifies 535 new loci associated with blood pressure traits. Nat. Genet..

[42] Mishra, M. K. et al. Comparative and functional genomic resource for mechanistic studies of human blood pressure-associated single nucleotide polymorphisms. *Hypertension*, 10.1161/HYPERTENSIONAHA.119.14109 (2020). 10.1161/HYPERTENSIONAHA.119.14109 PMC7035167 31902252

[43] Ovrehus MA (2019). Gene expression studies and targeted metabolomics reveal disturbed serine, methionine, and tyrosine metabolism in early hypertensive nephrosclerosis. Kidney Int. Rep..

[44] Lerman LO (2019). Animal models of hypertension: a scientific statement from the American Heart Association. Hypertension.

[45] Rapp JP (1982). Dahl salt-susceptible and salt-resistant rats. A review. Hypertension.

[46] Cowley AW (2008). Renal medullary oxidative stress, pressure-natriuresis, and hypertension. Hypertension.

[47] Geurts AM (2015). Maternal diet during gestation and lactation modifies the severity of salt-induced hypertension and renal injury in Dahl salt-sensitive rats. Hypertension.

[48] Palm F, Nordquist L (2011). Renal oxidative stress, oxygenation, and hypertension. Am. J. Physiol. Regulatory, Integr. Comp. Physiol..

[49] Welch WJ (2006). Intrarenal oxygen and hypertension. Clin. Exp. Pharmacol. Physiol..

[50] Hansell P, Welch WJ, Blantz RC, Palm F (2013). Determinants of kidney oxygen consumption and their relationship to tissue oxygen tension in diabetes and hypertension. Clin. Exp. Pharmacol. Physiol..

[51] Roman RJ (1990). Alterations in renal medullary hemodynamics and the pressure-natriuretic response in genetic hypertension. Am. J. Hypertens..

[52] Welch WJ, Baumgartl H, Lubbers D, Wilcox CS (2001). Nephron pO2 and renal oxygen usage in the hypertensive rat kidney. Kidney Int..

[53] Xie YW (1996). Role of endothelium-derived nitric oxide in the modulation of canine myocardial mitochondrial respiration in vitro. Implications for the development of heart failure. Circulation Res..

[54] Adler S, Huang H (2002). Impaired regulation of renal oxygen consumption in spontaneously hypertensive rats. J. Am. Soc. Nephrology: JASN.

[55] Lee H (2014). Increased mitochondrial activity in renal proximal tubule cells from young spontaneously hypertensive rats. Kidney Int..

[56] Kirchner KA (1990). Greater loop chloride uptake contributes to blunted pressure natriuresis in Dahl salt sensitive rats. J. Am. Soc. Nephrol..

[57] Roman RJ (1986). Abnormal renal hemodynamics and pressure-natriuresis relationship in Dahl salt-sensitive rats. Am. J. Physiol..

[58] Haque MZ, Ares GR, Caceres PS, Ortiz PA (2011). High salt differentially regulates surface NKCC2 expression in thick ascending limbs of Dahl salt-sensitive and salt-resistant rats. Am. J. Physiol. Ren. Physiol..

[59] Miyata N, Cowley AW (1999). Renal intramedullary infusion of L-arginine prevents reduction of medullary blood flow and hypertension in Dahl salt-sensitive rats. Hypertension.

[60] Evans LC (2015). Null mutation of the nicotinamide adenine dinucleotide phosphate-oxidase subunit p67phox protects the Dahl-S rat from salt-induced reductions in medullary blood flow and glomerular filtration rate. Hypertension.

[61] He X (2014). Ultrastructure of mitochondria and the endoplasmic reticulum in renal tubules of Dahl salt-sensitive rats. Am. J. Physiol. Ren. Physiol..

[62] Zheleznova NN (2012). Mitochondrial proteomic analysis reveals deficiencies in oxygen utilization in medullary thick ascending limb of Henle in the Dahl salt-sensitive rat. Physiol. Genomics.

[63] Wang Z, Sun Q, Sun N, Liang M, Tian Z (2017). Mitochondrial dysfunction and altered renal metabolism in Dahl salt-sensitive rats. Kidney Blood Press. Res..

[64] Rinschen, M. M. et al. Metabolic rewiring of the hypertensive kidney. *Sci. Signal*. **12**, 10.1126/scisignal.aax9760 (2019). 10.1126/scisignal.aax9760 PMC7273358 31822592

[65] Araujo M, Wilcox CS (2014). Oxidative stress in hypertension: role of the kidney. Antioxid. Redox Signal.

[66] Taylor NE, Maier KG, Roman RJ, Cowley AW (2006). NO synthase uncoupling in the kidney of Dahl S rats: role of dihydrobiopterin. Hypertension.

[67] Feng D (2012). Increased expression of NAD(P)H oxidase subunit p67(phox) in the renal medulla contributes to excess oxidative stress and salt-sensitive hypertension. Cell Metab..

[68] Taylor NE, Glocka P, Liang M, Cowley AW (2006). NADPH oxidase in the renal medulla causes oxidative stress and contributes to salt-sensitive hypertension in Dahl S rats. Hypertension.

[69] St-Pierre J, Buckingham JA, Roebuck SJ, Brand MD (2002). Topology of superoxide production from different sites in the mitochondrial electron transport chain. J. Biol. Chem..

[70] Tahara EB, Navarete FD, Kowaltowski AJ (2009). Tissue-, substrate-, and site-specific characteristics of mitochondrial reactive oxygen species generation. Free Radic. Biol. Med..

[71] Dikalov SI, Ungvari Z (2013). Role of mitochondrial oxidative stress in hypertension. Am. J. Physiol. Heart Circ. Physiol..

[72] Liang M (2011). Hypertension as a mitochondrial and metabolic disease. Kidney Int..

[73] Zhang A, Jia Z, Wang N, Tidwell TJ, Yang T (2011). Relative contributions of mitochondria and NADPH oxidase to deoxycorticosterone acetate-salt hypertension in mice. Kidney Int..

[74] Dikalova AE (2010). Therapeutic targeting of mitochondrial superoxide in hypertension. Circulation Res..

[75] Graham D (2009). Mitochondria-targeted antioxidant MitoQ10 improves endothelial function and attenuates cardiac hypertrophy. Hypertension.

[76] Zou L (2018). Knockout of mitochondrial voltage-dependent anion channel type 3 increases reactive oxygen species (ROS) levels and alters renal sodium transport. J. Biol. Chem..

[77] De Miguel C (2019). Uncoupling protein 2 increases blood pressure in DJ -1 knockout mice. J. Am. Heart Assoc..

[78] Tian Z (2009). Novel role of fumarate metabolism in dahl-salt sensitive hypertension. Hypertension.

[79] Hou E (2017). Malate and aspartate increase L-arginine and nitric oxide and attenuate hypertension. Cell Rep..

[80] Usa K (2017). Elevation of fumarase attenuates hypertension and can result from a nonsynonymous sequence variation or increased expression depending on rat strain. Physiol. Genomics.

[81] Taylor NE, Cowley AW (2005). Effect of renal medullary H_2_O_2_ on salt-induced hypertension and renal injury. Am. J. Physiol. Regulatory, Integr. Comp. Physiol..

[82] Zheng X (2019). Insufficient fumarase contributes to hypertension by an imbalance of redox metabolism in Dahl salt-sensitive rats. Hypertens. Res..

[83] You YH, Quach T, Saito R, Pham J, Sharma K (2016). Metabolomics reveals a key role for fumarate in mediating the effects of NADPH oxidase 4 in diabetic kidney disease. J. Am. Soc. Nephrology: JASN.

[84] Liang M, Knox FG (2000). Production and functional roles of nitric oxide in the proximal tubule. Am. J. Physiol. Regulatory, Integr. Comp. Physiol..

[85] Garvin JL, Herrera M, Ortiz PA (2011). Regulation of renal NaCl transport by nitric oxide, endothelin, and ATP: clinical implications. Annu Rev. Physiol..

[86] Miyata N, Zou AP, Mattson DL, Cowley AW (1998). Renal medullary interstitial infusion of L-arginine prevents hypertension in Dahl salt-sensitive rats. Am. J. Physiol..

[87] Chen PY, Sanders PW (1991). L-arginine abrogates salt-sensitive hypertension in Dahl/Rapp rats. J. Clin. Invest..

[88] Xue H (2019). Fumarase overexpression abolishes hypertension attributable to endothelial NO synthase haploinsufficiency in Dahl salt-sensitive rats. Hypertension.

[89] He W (2004). Citric acid cycle intermediates as ligands for orphan G-protein-coupled receptors. Nature.

[90] Vargas SL, Toma I, Kang JJ, Meer EJ, Peti-Peterdi J (2009). Activation of the succinate receptor GPR91 in macula densa cells causes renin release. J. Am. Soc. Nephrol..

[91] Toma I (2008). Succinate receptor GPR91 provides a direct link between high glucose levels and renin release in murine and rabbit kidney. J. Clin. Invest..

[92] Sadagopan N (2007). Circulating succinate is elevated in rodent models of hypertension and metabolic disease. Am. J. Hypertens..

[93] Wang L (2014). Analysis of metabolites in plasma reveals distinct metabolic features between Dahl salt-sensitive rats and consomic SS.13(BN) rats. Biochem. Biophys. Res. Commun..

[94] Liang M (2018). Epigenetic mechanisms and hypertension. Hypertension.

[95] Liu P (2018). Role of DNA de novo (de)methylation in the kidney in salt-induced. Hypertension.

[96] Wang Y, Liu X, Zhang C, Wang Z (2018). High salt diet induces metabolic alterations in multiple biological processes of Dahl salt-sensitive rats. J. Nutr. Biochem..

[97] Matsui R (2005). Glucose-6 phosphate dehydrogenase deficiency decreases the vascular response to angiotensin II. Circulation.

[98] Baillet A (2011). Coupling of 6-phosphogluconate dehydrogenase with NADPH oxidase in neutrophils: Nox2 activity regulation by NADPH availability. FASEB J..

[99] Spencer NY (2011). Control of hepatic nuclear superoxide production by glucose 6-phosphate dehydrogenase and NADPH oxidase-4. J. Biol. Chem..

[100] Wu L, Juurlink BHJ (2002). Increased methylglyoxal and oxidative stress in hypertensive rat vascular smooth muscle cells. Hypertension.

[101] Wang X, Desai K, Clausen JT, Wu L (2004). Increased methylglyoxal and advanced glycation end products in kidney from spontaneously hypertensive rats. Kidney Int..

[102] Chen X (2013). Carbonyl stress induces hypertension and cardio-renal vascular injury in Dahl salt-sensitive rats. Hypertension Res..

[103] Modan M (1985). Hyperinsulinemia. A link between hypertension obesity and glucose intolerance. J. Clin. Invest..

[104] DeFronzo RA, Cooke CR, Andres R, Faloona GR, Davis PJ (1975). The effect of insulin on renal handling of sodium, potassium, calcium, and phosphate in man. J. Clin. Investig..

[105] Kotchen TA, Zhang HY, Covelli M, Blehschmidt N (1991). Insulin resistance and blood pressure in Dahl rats and in one-kidney, one-clip hypertensive rats. Am. J. Physiol..

[106] Sechi LA (1997). Glucose metabolism and insulin receptor binding and mRNA levels in tissues of Dahl hypertensive rats. Am. J. Hypertens..

[107] Ogihara T (2002). High-salt diet enhances insulin signaling and induces insulin resistance in Dahl salt-sensitive rats. Hypertension.

[108] Kitada K (2017). High salt intake reprioritizes osmolyte and energy metabolism for body fluid conservation. J. Clin. Investig..

[109] Lin W (2018). High-salt diet affects amino acid metabolism in plasma and muscle of Dahl salt-sensitive rats. Amino Acids.

[110] Wang H (2020). Establishment of the circadian metabolic phenotype strategy in spontaneously hypertensive rats: a dynamic metabolomics study. J. Transl. Med..

[111] Crabos M (1997). Reduced basal NO-mediated dilation and decreased endothelial NO-synthase expression in coronary vessels of spontaneously hypertensive rats. J. Mol. Cell. Cardiol..

[112] Chou TC, Yen MH, Li CY, Ding YA (1998). Alterations of nitric oxide synthase expression with aging and hypertension in rats. Hypertension.

[113] Rajapakse NW (2012). Evidence that renal arginine transport is impaired in spontaneously hypertensive rats. Am. J. Physiol. Ren. Physiol..

[114] Racasan S (2004). Perinatal L-arginine and antioxidant supplements reduce adult blood pressure in spontaneously hypertensive rats. Hypertension.

[115] Matsuoka H (1996). Chronic L-arginine administration attenuates cardiac hypertrophy in spontaneously hypertensive rats. Hypertension.

[116] Ikeda Y, Saito K, Kim JI, Yokoyama M (1995). Nitric oxide synthase isoform activities in kidney of Dahl salt-sensitive rats. Hypertension.

[117] Chen PY, Sanders PW (1993). Role of nitric oxide synthesis in salt-sensitive hypertension in Dahl/Rapp rats. Hypertension.

[118] Szentiványi M (2002). Renal medullary nitric oxide deficit of Dahl S rats enhances hypertensive actions of angiotensin II. Am. J. Physiol. Regul. Integr. Comp. Physiol..

[119] Zhou MS (2001). L-Arginine improves endothelial function in renal artery of hypertensive Dahl rats. J. Hypertens..

[120] Fujii S, Zhang L, Igarashi J, Kosaka H (2003). L-arginine reverses p47phox and gp91phox expression induced by high salt in Dahl rats. Hypertension.

[121] Wu G, Morris SM (1998). Arginine metabolism: nitric oxide and beyond. Biochem. J..

[122] De Bandt JP (1995). Metabolism of ornithine, alpha-ketoglutarate and arginine in isolated perfused rat liver. Br. J. Nutr..

[123] Cynober L, Le Boucher J, Vasson M (1995). Arginine metebolism in mammals. J. Nutritional Biochem..

[124] Rajapakse NW, Mattson DL (2011). Role of L-arginine uptake mechanisms in renal blood flow responses to angiotensin II in rats. Acta Physiol..

[125] van de Poll MC, Soeters PB, Deutz NE, Fearon KC, Dejong CH (2004). Renal metabolism of amino acids: its role in interorgan amino acid exchange. Am. J. Clin. Nutr..

[126] Windmueller HG, Spaeth AE (1981). Source and fate of circulating citrulline. Am. J. Physiol..

[127] Cynober L (1994). Can arginine and ornithine support gut functions?. Gut.

[128] Haines RJ, Pendleton LC, Eichler DC (2011). Argininosuccinate synthase: at the center of arginine metabolism. Int. J. Biochem. Mol. Biol..

[129] Chien S-J (2014). Two different approaches to restore renal nitric oxide and prevent hypertension in young spontaneously hypertensive rats: l-citrulline and nitrate. Transl. Res.: J. Lab. Clin. Med..

[130] Koeners MP (2007). Maternal supplementation with citrulline increases renal nitric oxide in young spontaneously hypertensive rats and has long-term antihypertensive effects. Hypertension.

[131] Wu G, Fang YZ, Yang S, Lupton JR, Turner ND (2004). Glutathione metabolism and its implications for health. J. Nutr..

[132] Vasdev S, Singal P, Gill V (2009). The antihypertensive effect of cysteine. Int. J. Angiol..

[133] Chesney RW, Han X, Patters AB (2010). Taurine and the renal system. J. Biomed. Sci..

[134] Sun Q (2016). Taurine supplementation lowers blood pressure and improves vascular function in prehypertension: randomized, double-blind, placebo-controlled study. Hypertension.

[135] Ideishi M (1994). Taurine amplifies renal kallikrein and prevents salt-induced hypertension in Dahl rats. J. Hypertension.

[136] Trachtman H, Del Pizzo R, Rao P, Rujikarn N, Sturman JA (1989). Taurine lowers blood pressure in the spontaneously hypertensive rat by a catecholamine independent mechanism. Am. J. Hypertension.

[137] Mozaffari MS, Patel C, Abdelsayed R, Schaffer SW (2006). Accelerated NaCl-induced hypertension in taurine-deficient rat: role of renal function. Kidney Int..

[138] Armando I, Villar VA, Jose PA (2011). Dopamine and renal function and blood pressure regulation. Compr. Physiol..

[139] Caplin B (2012). Alanine-glyoxylate aminotransferase-2 metabolizes endogenous methylarginines, regulates NO, and controls blood pressure. Arteriosclerosis, Thrombosis, Vasc. Biol..

[140] De Miguel C, Lund H, Mattson DL (2011). High dietary protein exacerbates hypertension and renal damage in Dahl SS rats by increasing infiltrating immune cells in the kidney. Hypertension.

[141] Mattson DL, Meister CJ, Marcelle ML (2005). Dietary protein source determines the degree of hypertension and renal disease in the Dahl salt-sensitive rat. Hypertension.

[142] Hall JE, do Carmo JM, da Silva AA, Wang Z, Hall ME (2015). Obesity-induced hypertension: interaction of neurohumoral and renal mechanisms. Circulation Res..

[143] Ishizaka N (2010). Effects of the AT(1) receptor blocker losartan and the calcium channel blocker benidipine on the accumulation of lipids in the kidney of a rat model of metabolic syndrome. Hypertens. Res..

[144] Ding, W. et al. Osteopontin deficiency ameliorates Alport pathology by preventing tubular metabolic deficits. *JCI Insight* **3**, 10.1172/jci.insight.94818 (2018). 10.1172/jci.insight.94818 PMC5926939 29563333

[145] Chakraborty S (2018). Salt-responsive metabolite, beta-hydroxybutyrate, attenuates hypertension. Cell Rep..

[146] Mudaliar S, Alloju S, Henry RR (2016). Can a shift in fuel energetics explain the beneficial cardiorenal outcomes in the EMPA-REG OUTCOME study? A unifying hypothesis. Diabetes Care.

[147] Roman RJ, Fan F (2018). 20-HETE: hypertension and beyond. Hypertension.

[148] Hao CM, Breyer MD (2007). Physiologic and pathophysiologic roles of lipid mediators in the kidney. Kidney Int..

[149] Imig JD, Khan MA (2015). Cytochrome P450 and lipoxygenase metabolites on renal function. Compr. Physiol..

[150] Liang M (2007). Integrative pathway knowledge bases as a tool for systems molecular medicine. Physiol. Genomics.

[151] Williams AM (2018). Artificial intelligence, physiological genomics, and precision medicine. Physiol. Genomics.

[152] Ferrannini E (2017). Sodium-glucose co-transporters and their inhibition: clinical physiology. Cell Metab..

[153] Jose PA, Raj D (2015). Gut microbiota in hypertension. Curr. Opin. Nephrol. Hypertens..

